# Unique features of the transcriptional response to model aneuploidy in human cells

**DOI:** 10.1186/1471-2164-15-139

**Published:** 2014-02-18

**Authors:** Milena Dürrbaum, Anastasia Yurievna Kuznetsova, Verena Passerini, Silvia Stingele, Gabriele Stoehr, Zuzana Storchová

**Affiliations:** 1 Group Maintenance of Genome Stability, Max Planck Institute of Biochemistry, Am Klopferspitz 18, Martinsried 82152, Germany; 2 Department of Proteomics and Signal Transduction, Max Planck Institute of Biochemistry, Am Klopferspitz 18, Martinsried 82152, Germany; 3 Center for Integrated Protein Science Munich, Ludwig-Maximilian-University Munich, Munich 80336, Germany; 4 Current address: Gene Center, Department of Biochemistry, Ludwig-Maximilians-University Munich, Munich 81377, Germany

## Abstract

**Background:**

Aneuploidy, a karyotype deviating from multiples of a haploid chromosome set, affects the physiology of eukaryotes. In humans, aneuploidy is linked to pathological defects such as developmental abnormalities, mental retardation or cancer, but the underlying mechanisms remain elusive. There are many different types and origins of aneuploidy, but whether there is a uniform cellular response to aneuploidy in human cells has not been addressed so far.

**Results:**

Here we evaluate the transcription profiles of eleven trisomic and tetrasomic cell lines and two cell lines with complex aneuploid karyotypes. We identify a characteristic aneuploidy response pattern defined by upregulation of genes linked to endoplasmic reticulum, Golgi apparatus and lysosomes, and downregulation of DNA replication, transcription as well as ribosomes. Strikingly, complex aneuploidy elicits the same transcriptional changes as trisomy. To uncover the triggers of the response, we compared the profiles with transcription changes in human cells subjected to stress conditions. Interestingly, we found an overlap only with the response to treatment with the autophagy inhibitor bafilomycin A1. Finally, we identified 23 genes whose expression is significantly altered in all aneuploids and which may thus serve as aneuploidy markers.

**Conclusions:**

Our analysis shows that despite the variability in chromosome content, aneuploidy triggers uniform transcriptional response in human cells. A common response independent of the type of aneuploidy might be exploited as a novel target for cancer therapy. Moreover, the potential aneuploidy markers identified in our analysis might represent novel biomarkers to assess the malignant potential of a tumor.

## Background

Aneuploidy, or a change in cellular chromosome numbers, has profound effects on the physiology of all eukaryotic cells analyzed to date [1]. Aneuploid yeasts are characterized by slow growth, altered sensitivity to various stresses and increased genomic instability [2-4]. At the same time, aneuploidy drives genetic variability and cellular adaptation capacity in yeast [5,6]. Plants are in general more tolerant to gene dosage changes, yet aneuploidy often impairs their vigour and alters their phenotype [7]. Aneuploid mammals are rarely viable and the sporadic survivors are affected on multiple levels. In humans, aneuploidy is responsible for a substantial proportion of spontaneous abortions and the rare survivors with trisomy of chromosome 13, 18 and 21 (Patau, Edward and Down syndrome, respectively) are severely handicapped; only trisomy 21 is compatible with survival until adulthood [8]. Aneuploidy is also linked to cancer, as nearly 90% of solid tumors and 75% of hematopoietic cancers show abnormal chromosome dosage [9]. Recently it has been shown that the occurrence of aneuploid cells increases with aging [10] and an increased incidence of aneuploidy in the brain has been linked to neurodegenerative diseases [11].

The exact mechanisms underlying the detrimental effects of aneuploidy remain unclear, but it has been convincingly shown that they are caused by the expression of the extra genes on the supernumerary chromosome [3]. In most aneuploid cells the chromosome dosage changes lead to correlating changes in mRNA (e.g. [3,12-15]) as well as on protein levels [6,15,16]. These analyses further revealed that besides the increased abundance of transcripts and proteins originating from the aneuploid chromosome, the expression of multiple other genes is altered as well [3,13,15,17]. This is likely a consequence of two different phenomena. First, an increase in gene copy number of a transcription factor or other regulatory factor might affect transcription levels of genes on other chromosomes [18]. Second, specific pathways might be activated in a cellular response to aneuploidy. Recent attempts to uncover the consequences of aneuploidy suggest that aneuploidy indeed instigates a specific response in eukaryotic cells [3,15,19]. Haploid yeast strains carrying an additional chromosome (hereafter referred to as disomes) exhibit a common transcriptional signature that has been previously identified in budding yeast as a so called environmental stress response that is triggered upon various exogenous stresses, such as oxidative stress, heat shock or slow growth [3,20]. A similar response was identified in a study comparing transcriptome data from disomic and complex aneuploid strains of budding yeast, partial aneuploids of fission yeast, aneuploid *Arabidopsis thaliana* plant cells, mouse cell lines with Robertsonian translocations that lead to trisomies, and human cells from patients with trisomy syndromes [19]. Additionally, comparative transcriptomics and proteomics of model human trisomic and tetrasomic cells identified a common pattern in the transcriptional response to aneuploidy [15]. These studies pointed out similarities in the response to aneuploidy in most eukaryotes. At the same time, the results showed that the response in mammalian cells diverges from the response in other model organisms, because the correlation of the transcription changes in aneuploid mammalian cells with transcriptional changes in other aneuploid species is rather modest [19]. Moreover, as there is no equivalent of the environmental stress response identified so far in mammalian cells, it remains unclear what triggers the transcriptional changes.

To obtain a comprehensive insight into the changes specific for human cells, we evaluated in detail multiple tri- and tetrasomic cell lines as well as model complex aneuploid human cell lines with hypotetraploid karyotypes more similar to cancer cells. Remarkably, we found that the cellular responses to complex multichromosomal aneuploidy and trisomy or tetrasomy of one single chromosome closely correlate. As the identified pathway pattern resembles the cellular stress response, we compared the aneuploidy response to the transcriptional changes in human cells subjected to various stress conditions. Additionally, we have also identified 18 genes that were upregulated and 5 genes downregulated in all analyzed aneuploid cell lines and might thus serve as markers of aneuploidy. This is the first study which compares a variety of different human cell lines with aneuploidy of different types and origins. By uncovering a uniform aneuploidy response pattern our results outline the cellular consequences of an abnormal karyotype in human cells.

## Results

### Common cellular response to trisomy and tetrasomy in human cell lines

Recently, we established *de novo* human aneuploid cell lines that were derived from diploid and chromosomally stable cells by a micronuclei-mediated chromosome transfer [15]. We performed transcriptional analysis of the original diploid cell lines and their trisomic and tetrasomic derivatives (HCT116: trisomy of chromosome 3 – 3/3; tetrasomy of chromosome 5 - 5/4; RPE1: trisomy of chromosome 5 and 12 - 5/3 12/3, trisomy of chromosome 21 - 21/3, see Methods for further details) and calculated the aneuploid-to-diploid fold change for all detected mRNAs. Similarly, we calculated the relative aneuploid-to-diploid ratio to determine the transcriptional changes in trisomic human cells lines generated from the diploid colorectal adenocarcinoma cell line DLD1 by introduction of an extra copy of chromosome 3, 7 or 13 [13]. To identify the similarities and differences among the transcriptional profiles of the model aneuploid cell lines in response to aneuploidy, we used an algorithm called 2-dimensional annotation analysis that quantifies the relative up- and downregulation of cellular pathways. Hereby, abundance changes of all factors assigned to each pathway are compared to the overall abundance distribution and relative values are calculated [21]. Additionally, significantly up- and downregulated pathways were validated by the annotation enrichment tool DAVID, which employs a different algorithm for the analysis (data not shown) [22]. As expected, pathways altered and enriched in the 2-dimensional annotation analysis were also identified as enriched by DAVID in all cell lines.

**Figure 1:**
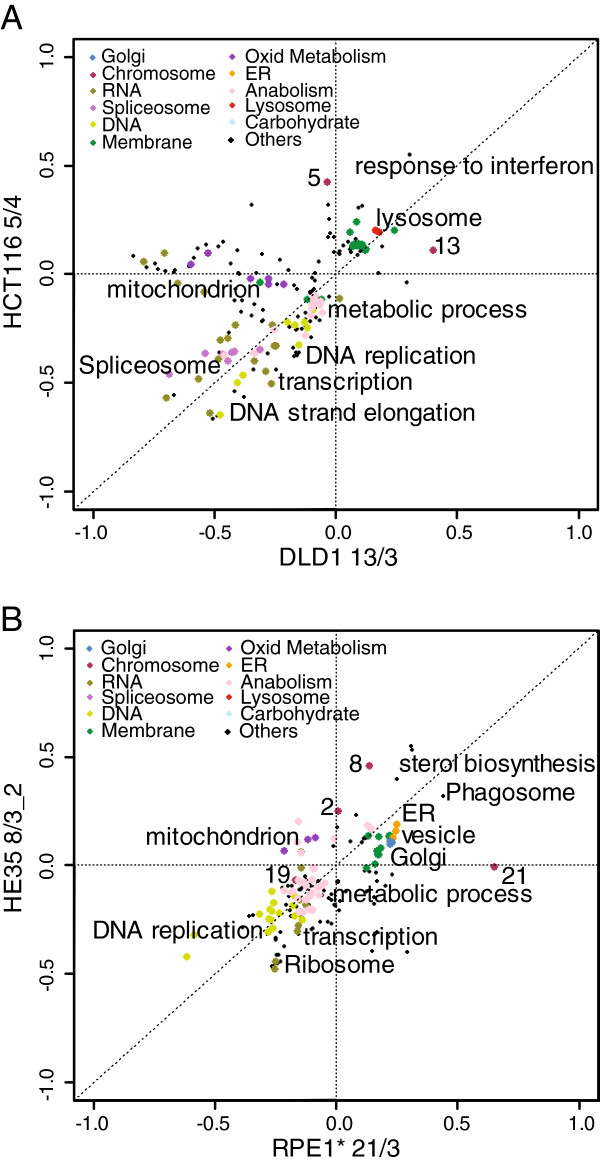
**Pathway alterations in tri- and tetrasomic cell lines. A**. 2-dimensional pathway analysis comparison of HCT116 with tetrasomy of chromosome 5 and DLD1 with trisomy of chromosome 13. Each dot represents an annotation as defined by GO and KEGG databases, color-coding marks related annotations as stated in the inset legend. The label “Others” indicates annotations that were not generalized in larger categories (see Methods). Examples of specific pathways categorized by the color- code are annotated. **B**. 2-dimensional pathway analysis comparison of HE35 (clone 2) with trisomy of chromosome 8 and RPE* with trisomy of chromosome 21. Asterisks indicate the cell lines with H2B-GFP.

Our analysis identified a specific pattern of pathways that are altered in all compared model trisomic and tetrasomic cell lines (Table 1). Among the most upregulated categories we identified the endoplasmic reticulum (ER), Golgi apparatus, lysosomes and vacuoles, membrane metabolism and the MHC protein complex and antigen processing. In contrast, DNA and RNA metabolic pathways – e.g. DNA replication, repair, transcription or RNA splicing - were significantly downregulated (Figure 1A, Additional file 1: Figure S1A). Remarkably, although similar pathways are up- and downregulated in all aneuploid cell lines, we noticed that the specific factors that significantly contribute to the differentiated regulation are variable. For example, the members of the proton oligopeptide cotransporter family SCL15 (Gene Ontology Cellular Component “membrane, lysosome, lytic vacuole”) show variable expression levels in the analyzed aneuploid cell lines: SLC15A3 is upregulated in all of the HCT116 derived aneuploid cell lines, but not in the RPE1 derived aneuploid cell lines, whereas SLC15A4 is upregulated only in HCT116 H2B-GFP 5/4 and SLC15A1 is upregulated in three out of seven aneuploid cell lines derived from HCT116 and RPE1 (Additional file 2: Table S1).

**Table 1:** Pathway classes recurrently and significantly altered in aneuploid cell lines

	**Replication**	**Transcription**	**Ribosome**	**Golgi**	**ER**	**Lysosomes**	**Membrane metabolism**	**Vacuole**	**Mitochondria**	**Spliceosome**	**MHC class proteins**
**HCT116 5/4**	-	-	-	+	+	+	+	+	-	-	+
**HCT116 3/3**	-	-	-	+	+	+	+	+	-	-	
HCT116 5/4*	-	-	-	+	+	+	(+)	+	-	/	+
**HPT1**	-	-	+	-	+	+	+	+	/	-	+
**HPT2**	-	-	-	+	+	+	+	+	+	-	+
**RPE1 12/3 5/3**	-	-	+	+	+	+	+	+	+	+	+
RPE1 21/3*	-	-	-	+	+	+	+	+	-	-	+
**DLD1 3/3**	-	-	-	+	/	/	+	+	-	-	/
**DLD1 7/3**	-	-	/	/	/	/	/	+	/	-	/
**DLD1 13/3**	-	-	-	+	/	/	+	+	-	-	/
**HE35 8/3_1**	+/-	-	-	-	- /	/	+/-	/	+	+	/
**HE35 8/3_2**	-	-	-	+	+	/	+/-	/	+/-	+	+
**HE35 8/3_3**	-	-/+	-	+	+	+	+/-	+	(+)	+	/

Asterisks indicate the cell lines expressing H2B-GFP.

/ = annotation not significantly altered.

+ = annotation category upregulated.

- = annotation category downregulated.

Interestingly, DLD1 with trisomy of chromosome 7 shows only partial overlap with the other aneuploid cell lines: pathways related to splicing and to DNA and RNA metabolism were similarly downregulated, and vacuole was upregulated, whereas additional changes in the transcription profile differed from the other trisomic and tetrasomic cell lines (Additional file 1: Figure S1B, Table 1). We also determined the response to aneuploidy in three clones with trisomy of chromosome 8 (here labeled as HE35 8/3_1, 2, and 3) derived from human primary embryonic fibroblasts HE35 by microcell-mediated chromosome transfer [17]. Using the 2-D annotation analysis algorithm, we observed similar changes in pathway regulations in the HE35-derived trisomies as in the other tri- and tetrasomic cell lines (Figure 1B, Additional file 1: Figure S1C, Table 1). Taken together, the results indicate that all the analyzed human cells with *de novo* created trisomies and tetrasomies elicit a nearly identical pattern of pathway changes regardless of the identity of the supernumerary chromosome and the cell line.

### Complex aneuploidy triggers the same pathway changes as low-complexity aneuploidy

**Figure 2:**
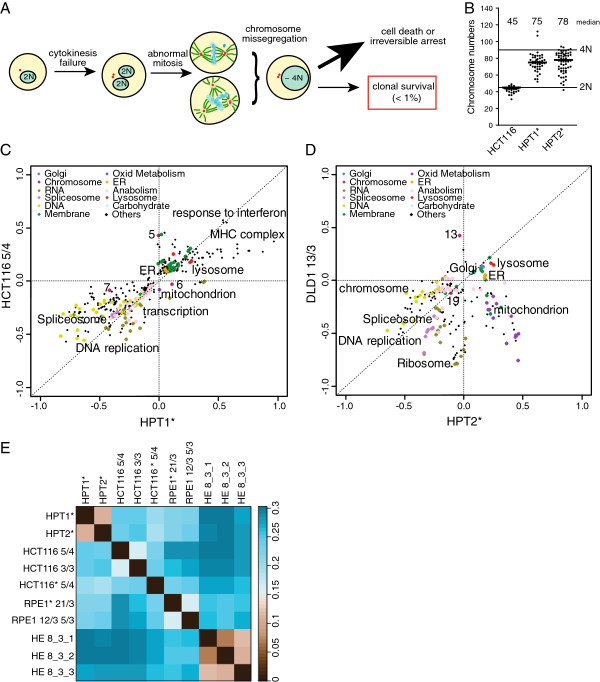
**Complex aneuploidy triggers similar pathway changes as trisomy or tetrasomy. A**. Schematic depiction of the construction of aneuploid cell lines with complex karyotype changes. **B**. Chromosome numbers in clonal cell lines originating from an unstable tetraploid intermediate. The numbers above the plot indicate the median chromosome number. **C**. 2-dimensional pathway analysis comparison of the complex aneuploid cell line HPT1* and HCT116 5/4. **D**. 2-dimensional comparison of complex aneuploid cell line HPT2* and DLD1 13/3. **E**. Distance matrix of the Spearman rank correlation of all analyzed aneuploid cell lines. Large distances present a low correlation and vice versa. The Spearman distance metric is defined as distanceSpearman = (1- correlationSpearman)/2 in the distanceMatrix function as part of the R Class Discovery package. The function calculates the distance of the correlation between column vectors, each of which represents different microarray experiments. Asterisks indicate the cell lines with H2B-GFP.

Next, we asked whether similar pattern of changes in gene expression could be observed in cells with complex aneuploidy. This type of karyotypic changes might more closely resemble the situation observed in tumors that often harbor multiple changes in chromosome numbers and structures, including deletions, translocations and amplifications [23]. To this end, we used cell lines derived from HCT116 that underwent a transient tetraploidy induced by cytokinesis failure (Figure 2A). As tetraploidy leads to catastrophic mitosis and chromosomal instability, the majority of cells die soon after the tetraploidization [9,24], and the chromosome numbers of the few survivors are remarkably altered (Figure 2B). We analyzed the transcriptional changes identified in two clonal survivors, cell lines HPT1 and HPT2, by calculating the fold change of the transcripts in comparison to the original HCT116. This first global analysis of the transcriptional response in human cells with model complex aneuploidy reproduced the pattern of pathway changes observed in the tri- and tetrasomic cell lines (Figure 2C,D, Additional file 1: Figure S1D, E, Table 1, Additional file 3). Importantly, the duration of the cell cycle of the complex aneuploid cell lines is indistinguishable from diploids (Additional file 1: Figure S2). Previously, it has been proposed that the transcriptional changes identified in aneuploid cells are caused by the slow growth, which is typical for model trisomic cell lines [3,14,15]. Thus, our finding indicates that the response to aneuploidy in mammalian cells is not always associated with a slower progression through the cell cycle, and thereby the slower proliferation cannot be the only cause of the identified transcriptional changes.

Distance matrix of the Spearman rank correlation of all analyzed cell lines confirmed the high similarity among the transcriptional changes triggered by aneuploidy (Figure 2E). We call the identified common pattern of transcriptional changes the Aneuploidy Response Pattern (ARP). Our results demonstrate that trisomy can serve as a valuable model for analysis of complex aneuploidy. Moreover, the identification of ARP suggests that chromosomal imbalance itself might be exploited as a novel target for cancer therapy regardless the type of aneuploidy and the mechanism by which it was generated.

### Comparison of the aneuploidy response pattern with transcriptional responses to stress stimuli

**Figure 3:**
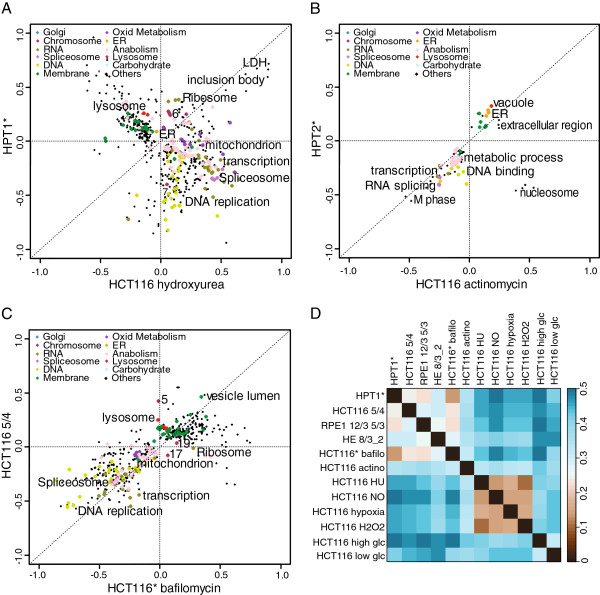
**Comparison of the Aneuploidy Response Pattern with transcriptional responses to stress stimuli. A**. 2-dimensional pathway analysis comparison of HCT116-derived complex aneuploid cell line HPT1* and HCT116 treated with hydroxyurea. **B**. 2- dimensional pathway analysis comparison of HCT116-derived complex aneuploid cell line HPT2* and HCT116 treated with actinomycin D. **C**. 2-dimensional pathway analysis comparison of HCT116 with tetrasomy of chromosome 5 and HCT116 grown in medium with bafilomycin A1 **D**. Distance matrix of the Spearman rank correlation of aneuploid cell lines and HCT116 grown in stress-inducing conditions. Asterisks indicate the cell lines with H2B-GFP. Abbreviations: bafilo = bafilomycin; actino = actinomycin; HU = hydroxyurea; NO = nitric oxide; H2O2 = hydrogen peroxide; glc = glucose.

What triggers the specific transcriptional response to aneuploidy in human cells? Current analysis in budding yeast suggests that mutations interfering with cell proliferation result in similar gene expression changes as aneuploidy [19]. Alternatively, the transcription changes may be triggered by cellular stress caused by the presence of an extra chromosome. Thus, we asked whether there are stress stimuli that trigger reprogramming of gene expression similar to the ARP. To this end, we compared the transcription profiles of HCT116 cells exposed to sub-lethal concentrations of hydrogen peroxide, nitric oxide, hydroxyurea, actinomycin D and bafilomycin A1, or grown under hypoxic conditions as well as in medium with either low or high glucose (for further details see Methods). Strikingly, the majority of the stressors triggered pathway changes that are remarkably different from the ARP (Figure 3A, Additional file 1: Figure S3). In contrast, we observed a partial similarity between the ARP and the transcriptional changes in HCT116 treated with actinomycin D, a polypeptide antibiotic that inhibits the activity of RNA polymerases (Figure 3B,D). The 2-D annotation analysis revealed that treatment with actinomycin results in downregulation of DNA- and RNA- metabolism as well as in upregulation of ER, membrane metabolism and lysosome. This might indicate that the downregulation of DNA- and RNA metabolism in aneuploid cell lines is due to transcriptional inhibition and its consequences. However, the overlap includes only a few genes with a low Spearman correlation coefficient and thus the distance of the Spearman rank correlation is higher compared to the distances between the aneuploid cell lines (Figure 3D). Remarkably, treatment with bafilomycin A1 that inhibits vacuolar-ATPases showed transcriptional changes nearly identical to the ARP (Figure 3C,D). Bafilomycin A1 impairs vesicle fusion [25] and thus inhibits the final steps of autophagy due to the failure of autophagosome-lysosome fusion. As in aneuploids, the transcriptional changes include downregulation of DNA and RNA metabolism, whereas membrane associated annotations are upregulated. In contrast, treatment with bafilomycin does not lead to upregulation of ER, Golgi or lysosomal pathways (Figure 3C). The similarity with transcriptional effects of autophagy-inhibiting drug suggests that one of the main consequences of aneuploidy in human cells is the overload of the autophagic pathway. Taken together, our findings imply that the transcriptional changes in response to aneuploidy differ from most of the common stress responses, but show shared features with response to conditions limiting autophagy and transcription.

### Markers of aneuploidy

**Figure 4:**
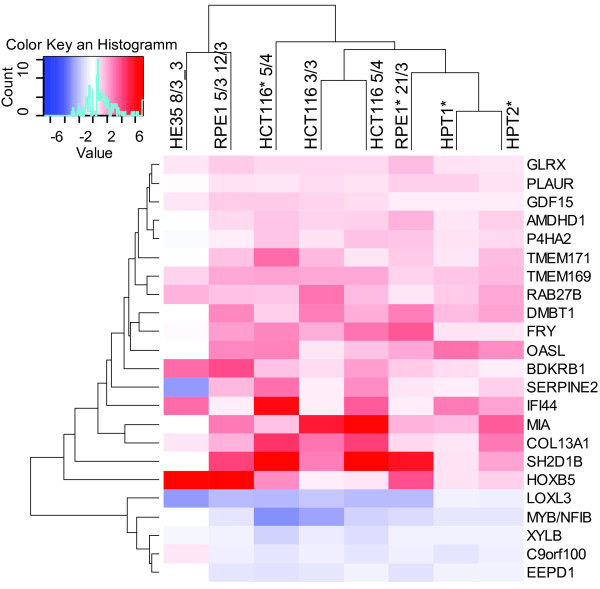
**Expression levels of the putative aneuploidy markers.** Heatmap of fold change ratios (aneuploid/diploid) of the 18 upregulated and 5 downregulated genes, hierarchical clustering of Euclidean distance. Asterisks indicate the cell lines with H2B-GFP.

**Figure 5:**
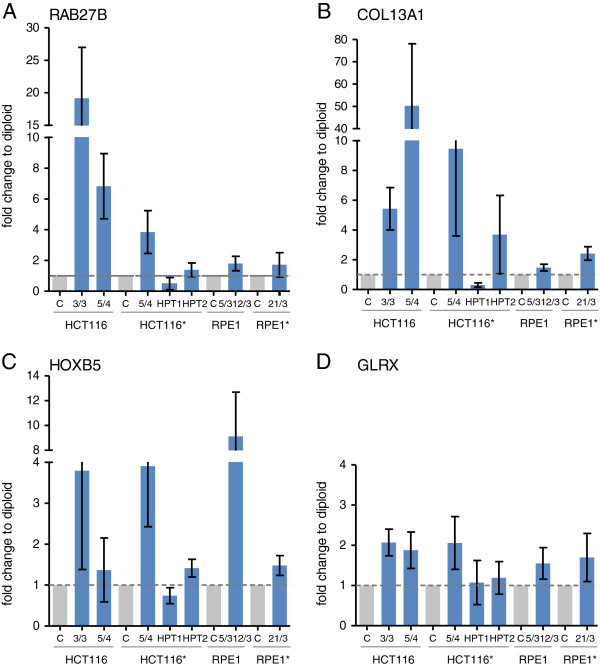
**Quantitative real-time PCR analysis of putative aneuploidy markers. A**. Fold change mRNA expression levels of COL13A1 quantified by real-time PCR. **B**. Fold change mRNA expression levels of RAB27B. **C**. Fold change mRNA expression levels of HOXB5. **D**. Fold change mRNA expression levels of GLRX. Asterisks indicate the cell lines expressing H2B-GFP.

Our results imply the existence of factors with recurrent expression changes triggered by aneuploidy that might serve as general markers of aneuploidy in human cells. To identify genes that are consistently up or downregulated in aneuploid cells, we merged all available datasets of aneuploid cell lines created in our laboratory. This yielded 18 genes whose expression is more than 1.4 fold increased and 5 genes whose expression is more than 1.4 fold decreased in all analyzed aneuploids (Figure 4, Table 2). The changes in mRNA levels are consistent with proteomics measurements in 7 out of 8 proteins for which the data are available [15] (Table 2). Importantly, expression of only one of these 23 genes (FRY) is similarly upregulated by the stress stimuli, suggesting that the recurrent expression changes are indeed in response to aneuploidy. Using quantitative real-time PCR we validated the expression levels of four of the identified upregulated genes in eight aneuploid cell lines and corresponding diploid controls (RAB27B, COL13A1, HOXB5, GLRX). This approach confirmed overexpression of all four genes in response to aneuploidy. Interestingly, none of the tested transcripts was confirmed in one of the complex aneuploid cell line, HPT1 (Figure 5). These candidate markers could potentially enable the discrimination of tumors with low frequency of aneuploid cells from tumors with high levels of aneuploidy and thus higher malignant potential. Further experiments will be required to determine whether the candidate markers can be used for clinical purposes for example by standard immunohistochemistry.

**Table 2:** List of consistently up- and downregulated transcripts (cut off 1.4 fold change)

	**Gene**	**Chromosome**	**Function**	**Protein levels**	**Link to cancer?**
1	PLAUR	19	Plasminogen activator, urokinase receptor	4/5	Ovarian and colorectal cancer
2	RAB27B	18	Secretory GTPase	4/4	Breast cancer
3	P4HA2	5	Procollagen-proline	4/6	Metastasis
4	FRY	13	Regulator of actin cytoskeleton	0/3	
5	BDKRB1	14	Bradykinine receptor	NA	Breast and lung cancer
6	HOXB5	17	Transcription factor	NA	Leukemia, ovarian carcinoma and others
7	GDF15	19	Growth differentiation factor	1/2	Ovarian and prostate cancer
8	OASL	12	2*'*-5*'*-oligoadenylate synthetase-like	NA	
9	SERPINE2	2	Serpine protease inhibitor	4/4	Metastasis
10	IFI44	1	Interferon-induced protein 44	NA	
11	AMDHD1	12	Protein with amidohydrolase domain	NA	
12	TMEM171	5	Transmembrane protein 171	NA	
13	GLRX	5	Glutaredoxin	4/4	Pancreatic cancer
14	TMEM169	2	Transmembrane protein 169	NA	
15	DMBT1	10	Membrane glycoprotein	NA	Multiple cancers
16	COL13A1	10	Collagen type XIII, alpha1	1/1	
17	SH2D1B	1	Signal transduction control	NA	
18	MIA		Melanoma inhibitory activity	NA	Neuronal tumors
					
1	XYLB	3	Xylulokinase homologue	NA	
2	LOXL3	2	Lysyl oxidase homologue	NA	
3	MYB/NFIB fusion	6	Myb – NFIB fusion	NA	
4	EEPD	7	Endonuclease/exonuclease/phosphatase domain containing	NA	
5	ARHGEF39	9	Rho guanine nucleotide exchange factor 39	NA	

The column “Protein levels” shows how often corresponding changes of the protein levels were identified by Stingele et al. [15] (number of cell lines with altered protein expression/number of cell lines where the protein was quantified; NA – proteomics data not available). The rows 1-18 show upregulated genes; the rows 1- 5 (below) show downregulated genes.

## Discussion

Significant recent progress in the analysis of aneuploidy in eukaryotes has revealed multiple novel features linked to aneuploidy such as growth defects, abnormal protein homeostasis and increased genomic instability. These features appear to be conserved among eukaryotes, an observation which is further supported by the finding that the transcriptional response to aneuploidy in different species remarkably correlates [19]. We analyzed in detail the transcriptional changes in aneuploid human cells by comparing the calculated aneuploid-to-diploid ratio. The comparison of eleven aneuploid model cell lines derived from four different diploid progenitor cell lines by either chromosome transfer of seven different chromosomes or by clonal propagation from unstable tetraploid progenitors revealed a striking similarity among the cell lines. The identified signature of pathways that was altered in all analyzed cell lines, which we term the aneuploidy response pattern - ARP - is characterized by upregulation of the ER and Golgi related pathways, lysosome and lytic vacuoles, MHC protein complex and antigen processing, whereas DNA and RNA metabolism and ribosome-related pathways were always downregulated (Figures 1, 2, Tables 1, 2). Our results reveal that the transcriptional changes to aneuploidy in human cells are not mediated by a canonical p53-dependent stress response, because DLD1 and its aneuploid derivatives carry mutant p53. A remarkable feature of the identified transcriptional changes is that although similar pathways are up- and downregulated in individual aneuploids, the individual genes whose expression is altered are often variable among the different cell lines. Furthermore, several pathways were up- or downregulated in the analyzed cells that were not recurrent and thus cannot be regarded as a general consequence of aneuploidy *per se*. These changes are probably specific to the type of extra chromosomes as well as to the clonal selection. We also show for the first time that the pathway changes in human cells with complex, hypotetraploid karyotypes correlate with the changes identified in simple trisomies. Thus, the analysis of model trisomic cell lines might provide important insights into the role of the more complex aneuploidy that is frequently found in cancer.

Two of the analyzed cell lines (trisomy 7 in DLD1 and one of the three clones of trisomy 8 in HE35) showed only a partial aneuploidy response pattern (Additional file 1: Figure S1B). There are several possible scenarios to explain this finding. First, loss of some chromosome parts or a mosaic aneuploid population could lead to a weakening of the ARP. However, the transcription levels of the transcripts coded on the extra chromosomes were elevated in these cell lines as expected, which excludes this possibility. Alternatively, features specific to the supernumerary chromosome can be responsible for the difference, such as in case of chromosome 7. This has been previously observed in budding yeast, where some disomes, e.g. disome of chromosome 1, do not trigger the environmental stress response pattern (ERS) otherwise observed in other disomic strains [3]. In support of this possibility is the observation that chromosome 7 is one of the few chromosomes which is more often gained than lost in chromosomally unstable cancer cells [26]. Thus, an extra copy of chromosome 7 likely represents a lesser burden for the cells than most of the other chromosomes. Finally, the deviation from the ARP might arise due to a mutation occurring during the clonal selection after the chromosome transfer. This is likely the case in HE35 8/3, where two clones correlate stronger with other aneuploids than the third trisomy 8 clone (Figure 2E). Despite these exceptions, our results show that aneuploidy triggers a uniform transcriptional response in human cells that is independent of the identity and quantity of the extra chromosomes and their combination as well as of the cell line type.

The ARP is characterized by strong downregulation of DNA and RNA metabolism, which correlates with the slow growth observed in nearly all aneuploid cells analyzed to date. Indeed, many factors required for DNA replication are less abundant, such as the heterohexameric replicative helicase MCM (Additional file 2: Table S1), and this may partially explain the previously observed slow progression through the S-phase [15]. However, the complex aneuploid cell lines progress through interphase at a rate closely matching the rate of the corresponding diploid cell lines and yet the levels of factors involved in replication are similarly decreased (Additional file 1: Figure S2). Therefore, we conclude that the proliferation impairment cannot be the only cause of ARP.

DNA repair is significantly downregulated, which might lead to increased accumulation of DNA damage. The abundance changes in the DNA replication and repair factors might explain how aneuploidy increases genome instability, as has been observed in yeast [4] as well as in human aneuploids (S.S., V.P., Z.S., unpublished results). Further research should elucidate what are the causes of the consistent downregulation of DNA metabolism pathways and what consequences it brings upon aneuploid cells.

Among the most upregulated pathways we observed the Golgi network, ER related pathways, lysosomes and lytic vacuoles, membrane metabolism and the MHC protein complex and antigen processing. The function of MHC protein complexes is to display fragments of proteins from within the cell to immune cells [27]. Currently we do not understand the reasons for the increased levels of the MHC protein complex transcripts in aneuploid cells. We hypothesize that increased protein expression and degradation elevates the peptide presentation by MHC complex, thus elevating the immunogenicity of aneuploid cells, similarly as it has been recently observed in murine aneuploid tumors [28]. In the future it will be important to confirm this observation on the protein level and to determine whether the MHC proteins are correctly localized and functional.

Elevated expression of ER related genes might suggest an expansion of the ER that was shown in budding yeast to occur in order to alleviate ER stress [29]. This phenomenon is accompanied by elevated lipid biosynthesis, but unchanged amounts of ER chaperones [29], which closely resembles the transcriptional changes observed in aneuploid cell lines. This suggests, together with the fact that mouse trisomic MEFs are more sensitive to the Hsp90 inhibitor 17-AAG [30], that aneuploid cells suffer from protein folding defects, most likely due to the saturation of the cellular folding capacity [31]. ER stress is often observed in various cancers, where it is usually attributed to tumor microenvironments characterized by hypoxia, nutrient limitation and low pH [32]. It will be interesting to test whether chromosomal copy number changes also contribute to the ER stress in cancer cells.

Previously, it was proposed that the transcriptional response to aneuploidy is indicative of stress and slow growth [1]. Therefore, we were interested whether treatment with stress inducing agents will elicit similar response as the presence of an extra chromosome. Since chromosome missegregation was shown to trigger oxidative stress [24,33], we analysed the transcriptional response to oxidative stress inducers nitric oxide and hydrogen dioxide. Aneuploidy was also recently linked to DNA damage and replication stress [34], therefore we analyzed the response to the replication inhibitor hydroxyurea and transcription inhibition by actinomycin D. Further, aneuploidy was shown to trigger proteotoxic stress and to activate autophagy [3,15,30,31]. Therefore, we compared the response to aneuploidy to the transcriptional changes elicited by cells grown under hypoxic conditions, which is known to result in energy and ER stress with subsequent proteotoxic stress. Similarly, we tested the transcriptional changes in cells where autophagy was inhibited, a condition known to trigger proteotoxic stress. The comparison with transcription profiles of cells grown on high or low glucose was of particular interest, because aneuploid cells show changes in the metabolic pathways and higher energy demands [3,14,15]. Remarkably, we found that none of the stress conditions triggers response similar to ARP.

The only significant exception was the striking similarity between the ARP and the transcriptional changes observed in cells treated with sub-lethal concentrations of bafilomycin A1 (Figure 3). Treatment with bafilomycin A1 leads to the accumulation of vesicles and membranes and reduces cell proliferation. The similarity of cellular response to inhibition of autophagy and to aneuploidy might suggest that aneuploidy inhibits autophagy. This however is not true as we previously documented by an observation that autophagic flux (the dynamic flow through autophagy) is in aneuploids as efficient as in diploids [15]. Inhibition of basal autophagy by bafilomycin A1 treatment causes proteotoxic stress [35]. Current data suggest that aneuploidy leads to proteotoxic stress as well [31]. We propose that the similarity between the transcriptional response to treatment with bafilomycin A1 and the ARP is because both reflect transcriptional changes in cells suffering from proteotoxic stress. In general, the effect of bafilomycin A1 further emphasizes the role of autophagy in mammalian aneuploids. Aneuploidy leads to activation of autophagy in human and mouse cells [15,30] and aneuploid cells are more sensitive to the autophagy inhibiting drug chloroquine [30]. The consistent upregulation of lysosomes and lytic vacuoles appears to be specific for mammalian cells as it was not observed in aneuploid yeasts and plants [19]. In this context it is interesting that there is nearly no correlation of ARP and transcriptional changes upon low glucose condition, which is known to trigger autophagy (Additional file 1: Figure S3). We believe that this is because autophagy in aneuploids is not activated by energy or nutrition deprivation, but by different, so far unidentified triggers [36]. Partial overlap of ARP was also observed with the response to treatment with the drug actinomycin D that represses transcription by RNA polymerase and, among other effects, leads to an imbalance of ribosomal subunits [37]. Transcription-related pathways are also downregulated in aneuploids, suggesting the possibility that some of the phenotypic features of aneuploid cells might be caused by transcriptional deficiency.

Our work has also allowed the identification of several genes that were consistently up- or downregulated in aneuploid cell lines, but not in the HCT116 cells under stress stimuli. Several of the identified factors have been previously linked to cancer (Table 2). For example the plasminogen activator and urokinase receptor PLAUR/UPAR is frequently overexpressed in ovarian and colorectal cancers, where it facilitates cell motility and metastatic potential and emerges as a marker of malignancy [38]. P4HA2 is a precursor of collagen and a metastasis marker [39] and HOXB5 is a homeobox transcription factor whose overexpression has been found in several malignant tumors [40]. Transcription of the TGFβ superfamily cytokine GDF15 (growth differentiation factor 15) is increased in multiple solid tumors, where it promotes tumor growth and confers resistance to several drugs such as bortezomib. Recent studies proposed GDF15 as a prognostic marker for ovarian and pancreatic carcinoma [41], two types of malignancies with frequent aneuploidy and chromosomal instability [9]. Glutaredoxin (GLRX) protects cells from oxidative stress and serves as a potential marker of malignancy in pancreatic carcinoma. Interestingly, some of the factors such as a xylulokinase homologue (XYLB), the 2′-5′-oligoadenylate synthetase like (OASL) and the interferon-induced protein (IFI44) have not been so far linked to cancer and may represent markers of aneuploidy that are not linked to malignancy.

Aneuploidy is associated with poor prognosis and increased drug resistance in tumors [42]. This suggests an interesting possibility that some of the genes that are expressed at higher levels in response to aneuploidy in non-transformed cells may subsequently enhance the malignant potential of these cells, whereas higher expression of others might represent a barrier for carcinogenesis. The expression of the marker genes, if confirmed in a wide range of aneuploid cell lines and in cancer cells, might provide an excellent tool for cancer biology and treatment, because it may allow distinguish tumors with high aneuploidy from predominantly diploid tumors. Moreover, pathways that are activated or inhibited by aneuploidy may serve as novel targets for cancer treatment. This has been recently demonstrated by the fact that aneuploid cells are more sensitive to drugs inhibiting HSP90, autophagy inhibitors and inhibitors of the AMPK kinase, which strongly correlates with the identified increased requirements for autophagy, protein folding and energy metabolism in aneuploid mammalian cells ([15,30] and this work). Further research should elucidate the efficacy of this approach. Our work for the first time identifies global changes in a broad spectrum of human aneuploid cell lines and may therefore help to generate new hypothesis for cancer treatments.

## Conclusions

In this study we identified a transcriptional aneuploid response pattern (ARP), a set of transcriptional changes, common in a broad range of human aneuploid model cell lines. This, for the first time, shows that complex aneuploidy, which is frequently found in cancer cells, exhibits the same transcriptional pathway changes as simple trisomy and tetrasomy. A general response to aneuploidy, as identified here in a variety of aneuploid model cell lines, might serve as a novel therapeutic target in cancer treatment. Further, we found 23 genes consistently deregulated in our model aneuploid cell lines. A confirmation of these markers in aneuploid cells might open new strategies for identifying aneuploid tumors. Since the fraction of aneuploid cells in tumor correlates with malignant potential and poor prognosis in cancer, simple and reliable biomarkers for aneuploidy may help for determining the appropriate cancer therapy.

## Methods

### Cell lines

Following model aneuploid cell lines were used for data analysis: parental cell line HCT116 (human colon carcinoma cell line): HCT116 3/3 (trisomy 3), HCT116 5/4 (tetrasomy 5) [43]; parental cell line HCT116 H2B-GFP: HCT116 5/4 (tetrasomy 5) [15], HPT1, HPT2 (hypertetraploids with complex karyotypes, A.Y.K. unpublished data); parental cell line RPE1 (human retinal pigment epithelial cell line, hTERT immortalized): RPE15/3 12/3 (trisomy 5, 12); parental cell line RPE1 H2B-GFP: RPE1 21/3 (trisomy 21) [15]; parental cell line DLD1 (human colon adenocarcinoma cell line): DLD1 3/3 (trisomy 3), DLD1 7/3 (trisomy 7), DLD1 13/3 (trisomy 13) [13], parental cell line HE35 (human embryonic cell line): HE35 8/3, clones 1–3 [17].

### Cell cultures

The HCT116- and RPE1- derived tri- and tetrasomic cell lines have been described previously [15]. The post- tetraploid cell lines HPT1 and HPT2 were generated by expansion of individual tetraploids formed by induced inhibition of cytokinesis through dihydrocytochalasin treatment. The DNA content was determined by flow cytometry, standard karyotyping, chromosome painting and array comparative genomic hybridization as in [15]. Cells were maintained in 10 cm dishes in Dulbecco’s Modified Eagle Medium (DMEM) at 37°C and 5% CO2. Culture medium was supplemented with growth factors from 5% Fetal Calf Serum (FCS) and 1% Penicillin/ Streptavidin to avoid bacterial growth and contamination. Specific antibiotics were added to the medium when necessary to maintain the supernumerary chromosomes. Cell lines were cultured at 70- 90% confluence for a maximum of 10 passages.

Material for transcription analysis was obtained at the earliest possible time point, after sufficient amount of cells was achieved, approximately 25 generations after the chromosome transfer or tetraploidization, respectively. Similar approach was taken for the analysis of trisomy 8 HE35-derived clones [17]. The mRNA of DLD1-derived aneuploids was isolated from multiple different passages [13].

### Cell growth analysis

Freshly cultured cells that carry H2B-GFP to fluorescently label the chromosomes were seeded 24 h before the experiment. Time laps movies were taken by imaging asynchronous cells in a 10 min or 4 min interval for 72 or 48 h, respectively. The time in interphase was measured as the time from nuclear envelope reformation to nuclear envelope break down.

### Quantitative real-time PCR

Total RNA was extracted with the RNeasy Mini Kit (Qiagen), treated with DNAse (recombinant DNase, Roche) and subsequently transcribed into cDNA (Transcriptor First Strand cDNA Synthesis Kit, Roche Diagnostics). Specific primers were designed using PrimerBlast; the sequences are listed below. Quantitative PCR was conducted using the Light Cycler 480 System (Roche Diagnostics) with the KAPA SYBR FAST master mix optimized for Roche Light Cycler 480 (KapaBiosystems). Absolute quantification with an external standard was performed and negative non-template controls were tested in all experiments. The specificity of the primer product amplification was confirmed in each run by melting curve analysis. mRNA expression was normalized to the control gene coding for ribosomal protein L30 (RPL30) and fold change to corresponding diploid mRNA expression was calculated. Primers: COL13A1 – forward: *GGGGGAAGCAGGACTAGATG*, reverse: *CCTGAAGCTCCGGGTAGTC,* RAB27B - forward: *TGCGGGACAAGAGCGGTTCCG*, reverse: *GCCAGTTCCCGAGCTTGCCGTT,* HOXB5 - forward: *TCCACAAATCAAGCCCTCCA*, reverse: *GTCCGGGCCATTTGGATAAC,* GLRX – forward: *AACGGTGCCTCGAGTCTTTA,* reverse: *CCTATGAGATCTGTGGTTACTGC.*

### mRNA expression analysis by microarrays

Genome-wide expression profiling of HCT116- and RPE1- derived aneuploid cells lines was conducted in three replicates by IMGM laboratories GmbH (Martinsried, Germany) as previously described [15]. cDNA was hybridized on Agilent Whole Human Genome Oligo microarrays (4x44K format) for HCT116 diploid and HCT116 5/4, or Agilent SurePrint G3 Human GE microarrays (8x60K) for the other cell lines according to a One-Color based hybridization protocol. Raw data was background normalized. The data has been deposited in the NCBI Gene Expression Omnibus (GEO, http://www.ncbi.nlm.nih.gov/geo/) accessible through GEO Series accession number GSE47830 and GSE47836.

Microarray data of trisomic and diploid colorectal cancer cell lines DLD1 [13] were kindly provided by Thomas Ried (National Institutes of Health, Bethesda, Maryland, USA). All other mRNA expression data were obtained from NCBI’s Gene Expression Omnibus (http://www.ncbi.nlm.nih.gov/geo/). HCT116 cell line grown under high and low glucose conditions: accession number GSE31084; HCT116 after stress treatment: accession number GSE3176; HCT116 cell line treated with actinomycin D: with GSE12459, human embryonic diploid and trisomic cells: accession number GSE28076.

### Microarray normalization

Bioinformatics analysis of the microarray data was performed using Perseus (1.2.6.16) as part of the MaxQuant Software Package [21] and R on the free open source integrated development environment R Studio. The gBGSubSignal (green Background Subtracted Signal) from background-normalized cDNA microarray data was used for further normalization. The background-subtracted raw intensities were log2 transformed and global normalization of the log transformed raw data was performed by subtracting the median of the overall signal intensities for one experiment from each signal in this experiment. Probe sets for one gene were summarized by taking the median. The median of replicative probes signal intensities was calculated for each cell line. For comparison of each aneuploid cell line with the corresponding diploid cell line gene, expression fold change ratios were calculated.

### Data analysis

The Student’s *t*-test was performed to verify the statistical significance of the fold change in mRNA expression between the signal intensities of aneuploid and diploid cell line. To correct the test statistics for multiple comparisons, false discovery rate control of the p-values was applied. Both local and frequent FDR were calculated with the “fdrtool” package [44]. For further analysis, a fold change cut off 1.4 in mRNA expression was applied. To investigate the correlation between the cell lines, a distance matrix of the Spearman rank correlation coefficient was calculated in R using the “ClassDiscovery” package in the OOMPA library and “mclust” package. Distance of the correlation was visualized in a colored matrix in the “spatstat” package. To investigate a global response to aneuploidy on gene level, data sets were filtered for those genes more than 1.4 fold up- or downregulated in all HCT116 and RPE1 derived cell lines. The resulting gene lists were analyzed for their function and relation using the DAVID functional annotation tool [22], KEGG pathway and the NCBI Gene database.

### Pathway enrichment analysis

Pathway enrichment analysis was conducted in Perseus using the 2-dimensional annotation enrichment tool [21]. Thereby, enriched GO and KEGG annotations were identified by testing whether genes in an annotated category have a significant preference to be altered compared to the global fold change data distribution. Significance was tested by a *t*-test followed by a false discovery rate correction in the Benjamini-Hochberg procedure (FDR cut off 0.02). Expression values for significant enriched annotations were summarized in an annotation score from -1 to 1 to represent levels of up- or downregulation. Significant and enriched annotations were summarized in larger, general categories for visualization purposes. 2-dimensional annotations enrichments of two cell lines were plotted in R. Highly up- or downregulated pathways were cross validated by submitting the fold change cut off datasets to the annotation enrichment tool DAVID [22].

### Statistical analysis

Commonly used R packages were “lattice” [45], “genefilter” [46], “fdrtool” [44] and “calibrate” [47]. In addition, Perseus (1.2.6.16) as part of the MaxQuant Software Package [21] was used for microarray analysis. All statistically evaluated experiments were performed in at least three independent biological replicates. The final plots were prepared in GraphPad Prism 5 software or R.

### Availability of supporting data

The data sets supporting the results of this article are available in the NCBI Gene Expression Omnibus repository (http://www.ncbi.nlm.nih.gov/geo/) with the accession numbers GSE47830 and GSE47836.

## Competing interests

The authors declare that they have no competing interests.

## Authors’ contributions

ZS initiated the study. AYK, SS and VP generated the cell lines and prepared the samples for analysis. MD performed the bioinformatics analysis and the quantitative RT PCR. MD, AYK, GS and ZS discussed and designed the data analysis strategies. ZS wrote the manuscript. All authors read and discussed the final manuscript.

## Supplementary Material

Additional file 1: Figure S1 Significantly altered pathways in aneuploid model cell lines. **Figure S2**. Time in interphase of the HCT116*- derived cell lines. **Figure S3**. Significantly altered pathways in response to stress stimuli compared to the ARP. Click here for file

Additional file 2 Dataset 1 Gene expression data of HCT116 and RPE1 derived aneuploid cell lines compared to the diploid cell lines. Click here for file

Additional file 3 Dataset 2 Data derived from the 2- dimensional annotation analysis. Click here for file
