# Supplementary material for: Unique features of the transcriptional response to model aneuploidy in human cells
Source: BMC Genomics. 2014 Feb 18;15:139. doi: 10.1186/1471-2164-15-139 (PMC3932016; doi:10.1186/1471-2164-15-139)
Supplement: Additional file 1: Figure S1 — Significantly altered pathways in aneuploid model cell lines. Figure S2. Time in interphase of the HCT116*- derived cell lines. Figure S3. Significantly altered pathways in response to stress stimuli compared to the ARP. [file 1471-2164-15-139-S1.pdf]

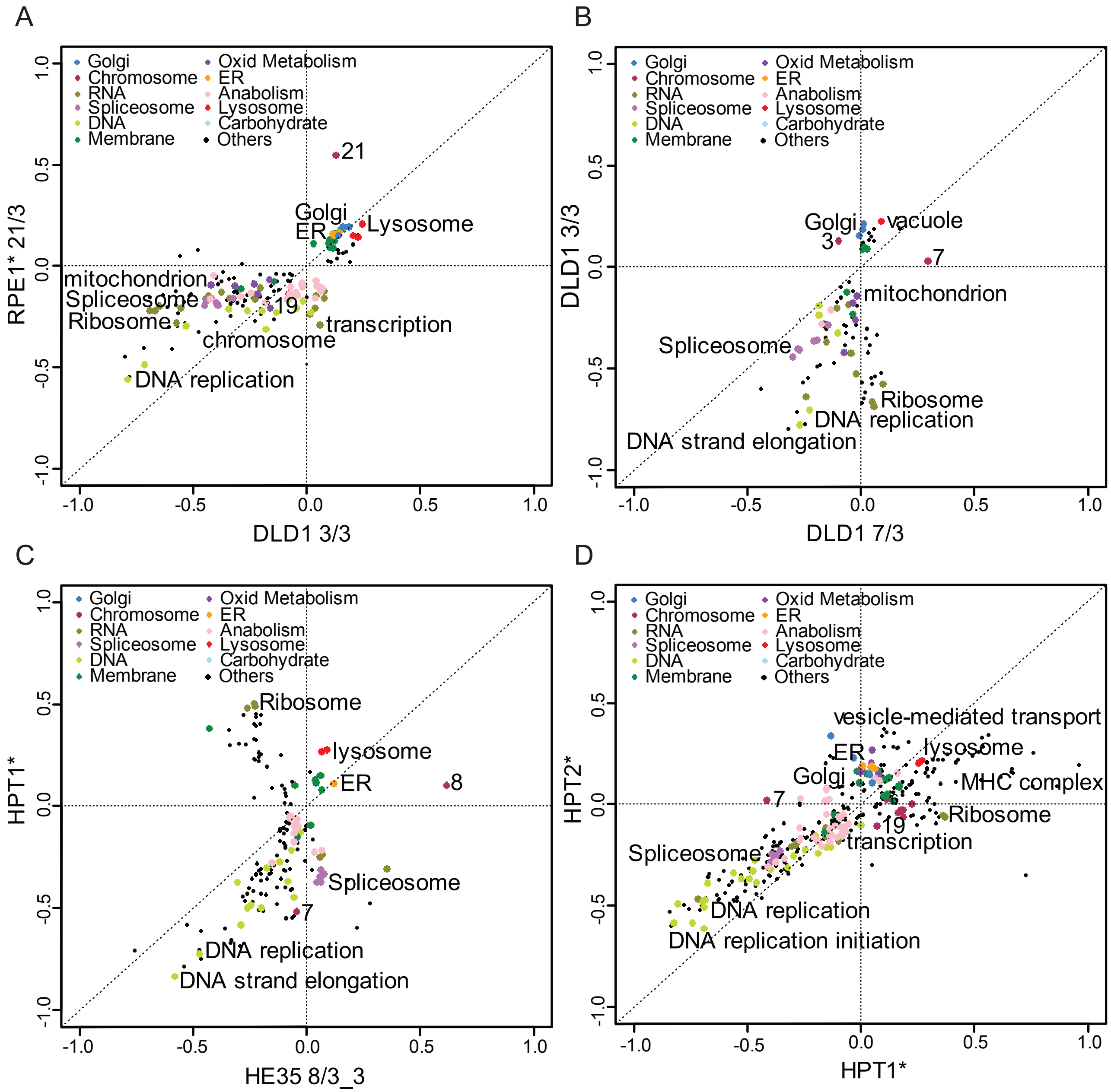

Supplementary Figure 1
Dürrbaum et al.

E

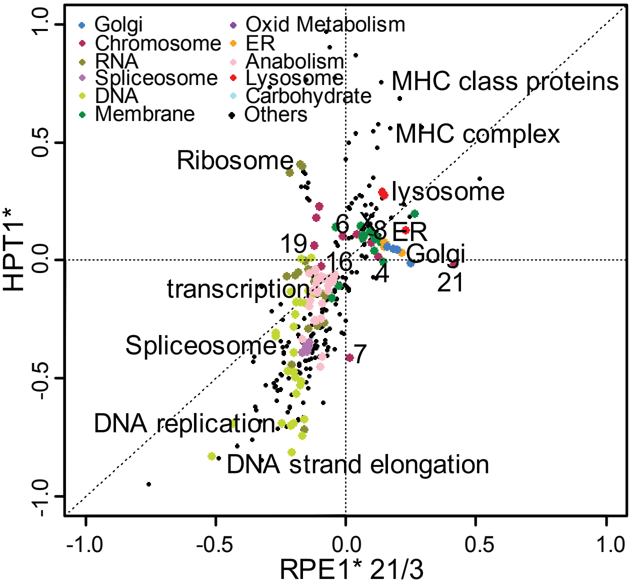

Figure S1. Significantly altered pathways in aneuploid model cell lines.
2- dimensional pathway analysis comparison of **A.** RPE1* with trisomy of chromosome 21 and DLD1 with trisomy of chromosome 3. **B.** DLD1 with trisomy of chromosome 13 and DLD1 with trisomy of chromosome 7. **C.** HCT116- derived complex aneuploid cell line HPT1* and HE35 with trisomy of chromosome 8, clone 3. **D.** HCT116- derived complex aneuploid cell line HPT2* and HPT1*. **E.** HCT116- derived complex aneuploid cell line HPT1* and RPE* with trisomy of chromosome 21. Asterisks indicate the cell lines with H2B-GFP.

Supplementary Figure 1
Dürrbaum et al.

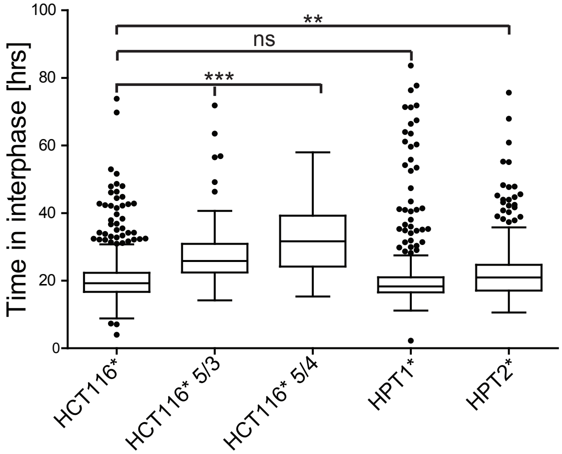

Figure S2. Time in interphase of the HCT116*- derived cell lines.
Time in interphase of the HCT116* derived cell lines with tri- and tetrasomy of chromosome 5 and with complex aneuploidy (HPT1*, HPT2*) compared to the diploid HCT116* cell line. Significance was calculated by applying one-way ANOVA test with Bonferroni´s correction for multiple comparisons (ns= P>0.05, *= P ≤ 0.05, ** = P ≤ 0.01, *** = P ≤ 0.001).
Asterisks indicate the cell lines with H2B-GFP.

Supplementary Figure 2
Dürrbaum et al.

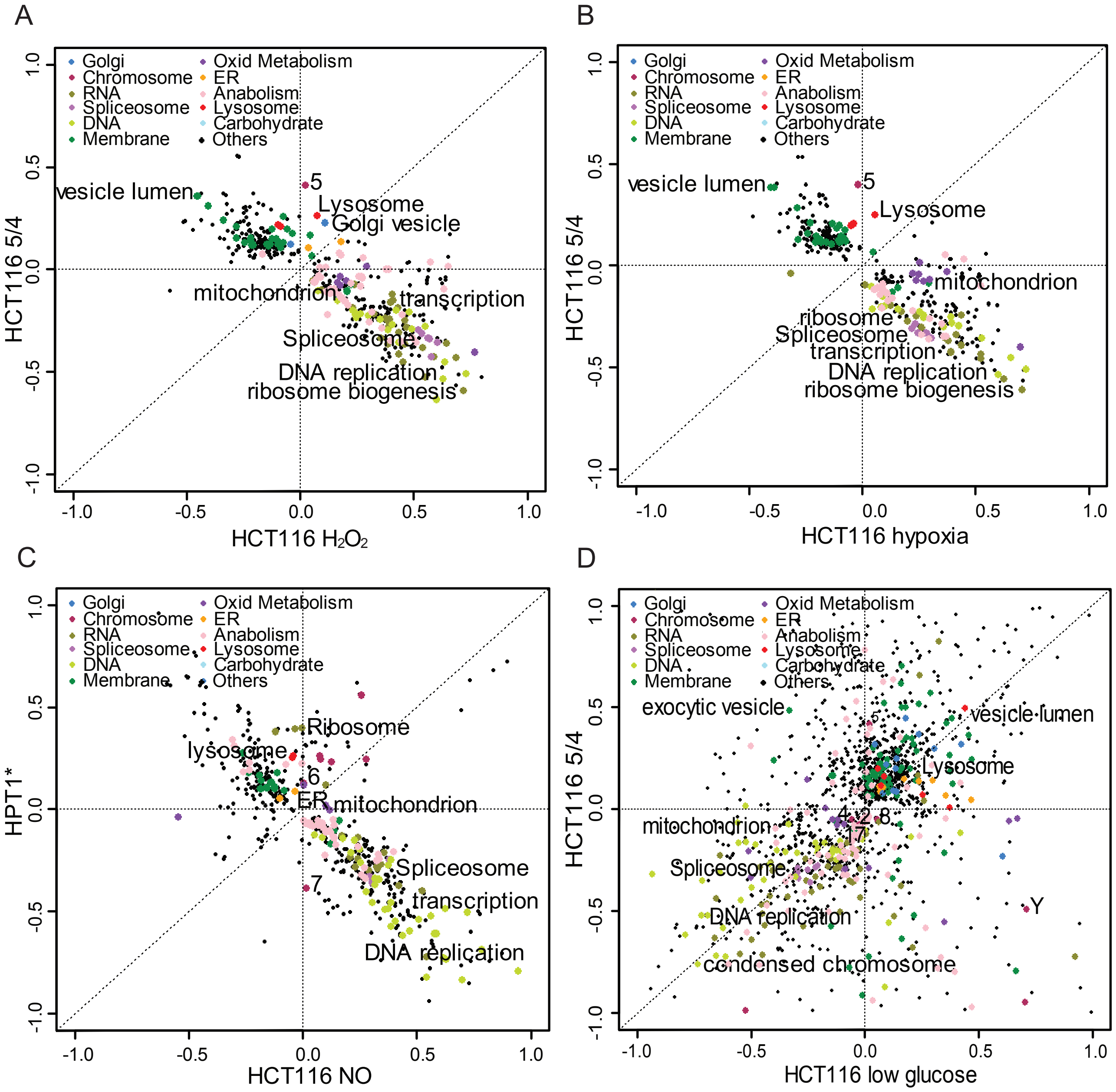

Supplementary Figure 3
Dürrbaum et al.

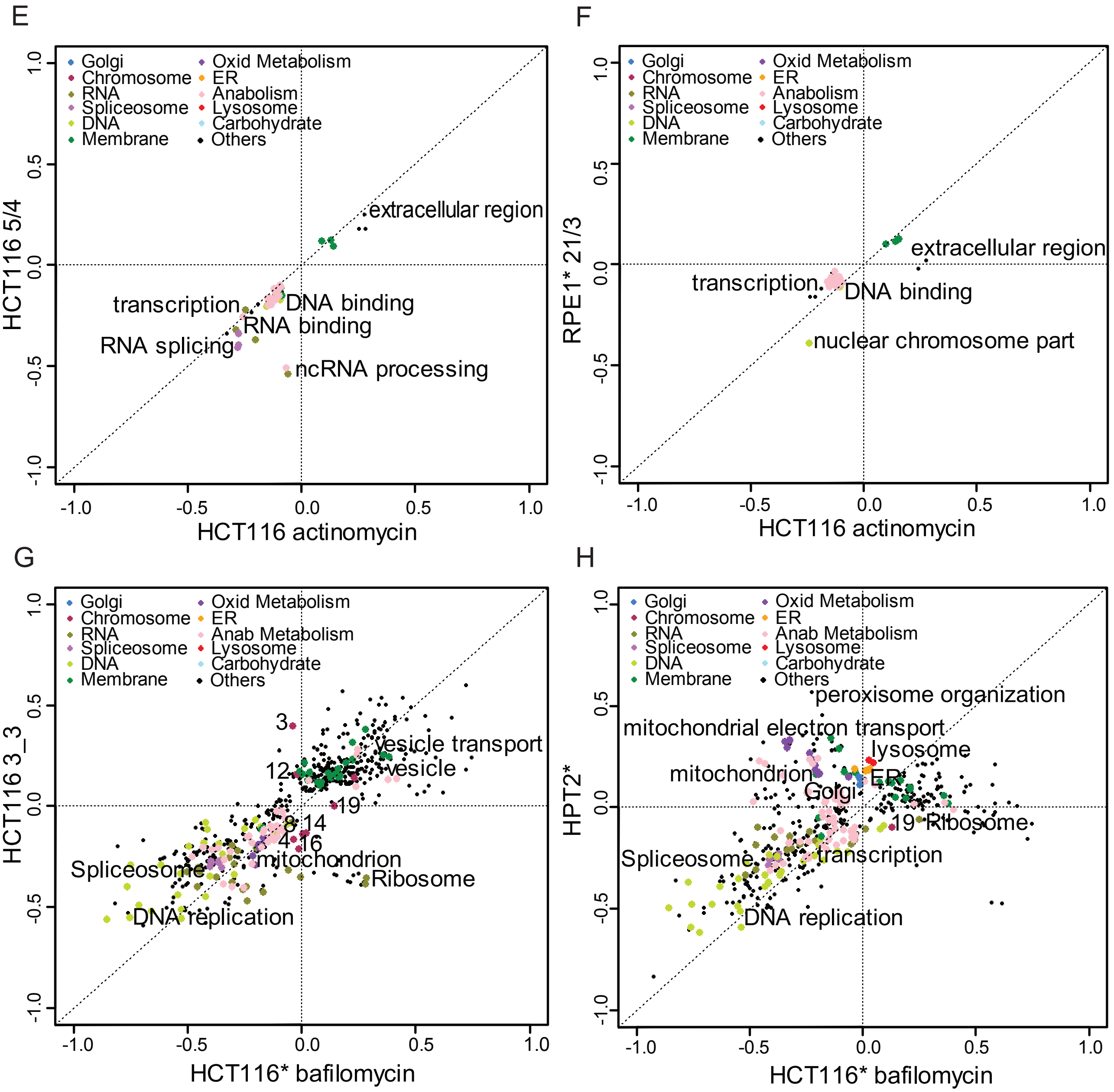

Figure S3. Significantly altered pathways in response to stress stimuli compared to the ARP. 2- dimensional pathway analysis comparison of **A.** HCT116 with tetrasomy of chromosome 5 and HCT116 treated with hydrogen peroxide (H2O2). **B.** HCT116 with tetrasomy of chromosome 5 and HCT116 grown under hypoxic conditions. **C.** HCT116- derived complex aneuploid HPT1* and HCT116 treated with nitric oxide (NO). **D.** HCT116 with tetrasomy of chromosome 5 and HCT116 grown in medium with low glucose. **E.** HCT116 with tetrasomy of chromosome 5 and HCT116 treated with actinomycin D. **F.** RPE1 with trisomy of chromosome 21 and HCT116 treated with actinomycin D. **G.** HCT116 with trisomy of chromosome 3 and HCT116 treated with bafilomycin. **H.** HCT116- derived complex aneuploid HPT2* and HCT116 treated with bafilomycin.
Asterisks indicate the cell lines with H2B-GFP.
